# Role of Muscarinic Acetylcholine Receptors in Serial Feature-Positive Discrimination Task during Eyeblink Conditioning in Mice

**DOI:** 10.1371/journal.pone.0147572

**Published:** 2016-01-25

**Authors:** Md. Ashrafur Rahman, Norifumi Tanaka, Koji Usui, Shigenori Kawahara

**Affiliations:** 1 Graduate School of Innovative Life Science, University of Toyama, 3190 Gofuku, Toyama, 930–8555, Japan; 2 Graduate School of Science and Engineering, University of Toyama, 3190 Gofuku, Toyama, 930–8555, Japan; Tokai University, JAPAN

## Abstract

We investigated the role of muscarinic acetylcholine receptors (mAChRs) in eyeblink serial feature-positive discrimination learning in mice using the mAChR antagonist. A 2-s light cue was delivered 5 or 6 s before the presentation of a 350-ms tone paired with a 100-ms periorbital electrical shock (cued trial) but not before the tone-alone presentation (non-cued trial). Mice received 30 cued and 30 non-cued trials each day in a random order. We found that saline-injected control mice were successfully discriminating between cued and non-cued trials within a few days of conditioning. The mice responded more frequently to the tone in cued trials than in non-cued trials. Analysis of conditioned response (CR) dynamics revealed that the CR onset latency was shorter in cued trials than in non-cued trials, despite the CR peak amplitude not differing significantly between the two conditions. In contrast, scopolamine-injected mice developed an equal number of CRs with similar temporal patterns irrespective of the presence of the cue during the 7 days of conditioning, indicating in a failure to acquire conditional discrimination. In addition, the scopolamine administration to the control mice after they had successfully acquired discrimination did not impair the conditional discrimination and expression of pre-acquired CR. These results suggest that mAChRs may play a pivotal role in memory formation in the conditional brain state associated with the feature cue; however they are unlikely to be involved in the development of discrimination after conditional memory had formed in the serial feature-positive discrimination task during eyeblink conditioning.

## Introduction

Classical eyeblink conditioning is one of the best models of learning for studying the interaction between higher and lower levels of the nervous system [1–3]. While many studies have focused on the underlying neural substrates for standard delay eyeblink conditioning, which essentially depends on the cerebellum and brainstem [4–6], the role of higher brain regions, such as the hippocampus and medial prefrontal cortex have also been under intense experimental scrutiny [7–14]. One of the proposed roles of higher brain regions in eyeblink conditioning is a strong top-down modulation of the ongoing input-output relationships in the lower regions of brain. This view is supported by studies of conditional discrimination tasks in patients with amnesia with temporal lobe lesions [15, 16]. Similar kinds of conditional discrimination tasks, also known as serial feature-positive discrimination or occasion setting [2], have been used in studies of rabbit eyeblink conditioning [17–21]. During serial feature-positive discrimination tasks, animals receive randomly alternating reinforced and non-reinforced presentations of the conditioned stimulus (CS). In reinforced trials, the feature conditional cue and the target CS are serially presented with or without a temporal gap, followed by the unconditioned stimulus (US), while in non-reinforced trials, the target CS is presented alone, without the feature cue or US. The animals learn to differentiate the conditioned response (CR) to CSs in reinforced and non-reinforced trials based on the presence/absence of the preceding feature stimulus.

Recently we investigated the modulation of hippocampal local field potentials during the serial feature-positive discrimination task in rat eyeblink conditioning and found a significant correlation between an increase in the relative power of hippocampal theta oscillations after the light cue and a subsequent expression of the CR on a trial-to-trial basis [22]. This strongly suggested a hippocampal involvement in the top-down modulation of the CR in this conditional discrimination task. Although the effect of hippocampal lesions on serial feature-positive discrimination has not been examined, the hippocampal contribution is consistent with the impairment of the conditional discrimination in amnesic patients [15, 16], as well as with the crucial involvement of the hippocampus in the simultaneous feature-positive discrimination, during which the light feature and tone targets were presented simultaneously [23].

It is well known that the hippocampus is innervated by cholinergic inputs originating from the medial septum [24] and is enriched with muscarinic acetylcholine receptors (mAChRs) [25]. The crucial role of mAChRs in learning and memory have been studied by using the mAChR antagonist scopolamine in various types of hippocampus-dependent learning tasks [26–28], including eyeblink conditioning in rabbits [29] and mice [30]. In addition, the role of mAChRs in attentional process has also been reported in rats [31] and humans [32]. Therefore, it is likely that mAChRs play an important role in the serial feature-positive discrimination in eyeblink conditioning, which is thought to place a much higher cognitive demand for memory formation and attentional power than the simple delay eyeblink conditioning, which does not necessary involve higher brain regions [5, 6].

In the present study, we investigated the role of mAChRs in the serial feature-positive discrimination task in mouse eyeblink conditioning by using scopolamine. We found that a systemic administration of scopolamine impaired the acquisition of the conditional discrimination, but did not affect the performance of the pre-acquired conditional discrimination. These observations indicated that mAChRs play a role in the formation of memory for the conditional discrimination rather than the discriminative performance based on the conditional cue.

## Materials and Methods

### Animals and ethics statement

Sixteen male 8-week-old C57Bl/6 mice were purchased from Japan SLC, Inc. (Hamamatsu, Shizuoka, Japan), housed in standard plastic cages on a 12/12-h light/dark cycle, and given free access to food and water. The room temperature was maintained at 24 ± 2°C. Mice were blindly divided into the control saline group and scopolamine groups (n = 8 each). Scopolamine hydrobromide (Tocris Bioscience, Ellisville, MO, USA) was administered intraperitoneally at a dose of 1.0 mg/kg in a solution volume of 5 mL per kg bodyweight. Equivalent volumes of saline were administered to control animals. The experiments were performed during the light period. All the experimental procedures were carried out in accordance with the NIH Guide for the Care and Use of Laboratory Animals and approved by the Experimental Animal Committee of the University of Toyama (A2014ENG-9). Throughout the experiments, all efforts were made to minimize the use of animals and to optimize their comfort.

### Surgical procedures

Animals were anesthetized with ketamine (80 mg/kg, i.p.; Sankyo, Tokyo, Japan) and xylazine (20 mg/kg, i.p.; Bayer, Tokyo, Japan). Four Teflon-coated stainless-steel wires (140 μm in diameter, A-M Systems, Sequim, WA, USA) were subcutaneously implanted in the left upper eyelid. Two of the four wires were used to record electromyograms (EMGs) for the CR detection and the other two delivered electrical shocks as unconditional stimuli (US). The connector pins soldered to the wires prior to the surgery were fixed to the skull with dental acrylic resin and stainless steel screws. Following the surgical procedure, mice were injected with ampicillin (100 mg/kg, i.p.; Meiji Seika, Tokyo, Japan), placed into a warm cage until they moved voluntarily, and then returned to their home cage.

### Conditioning procedure

**Fig 1:**
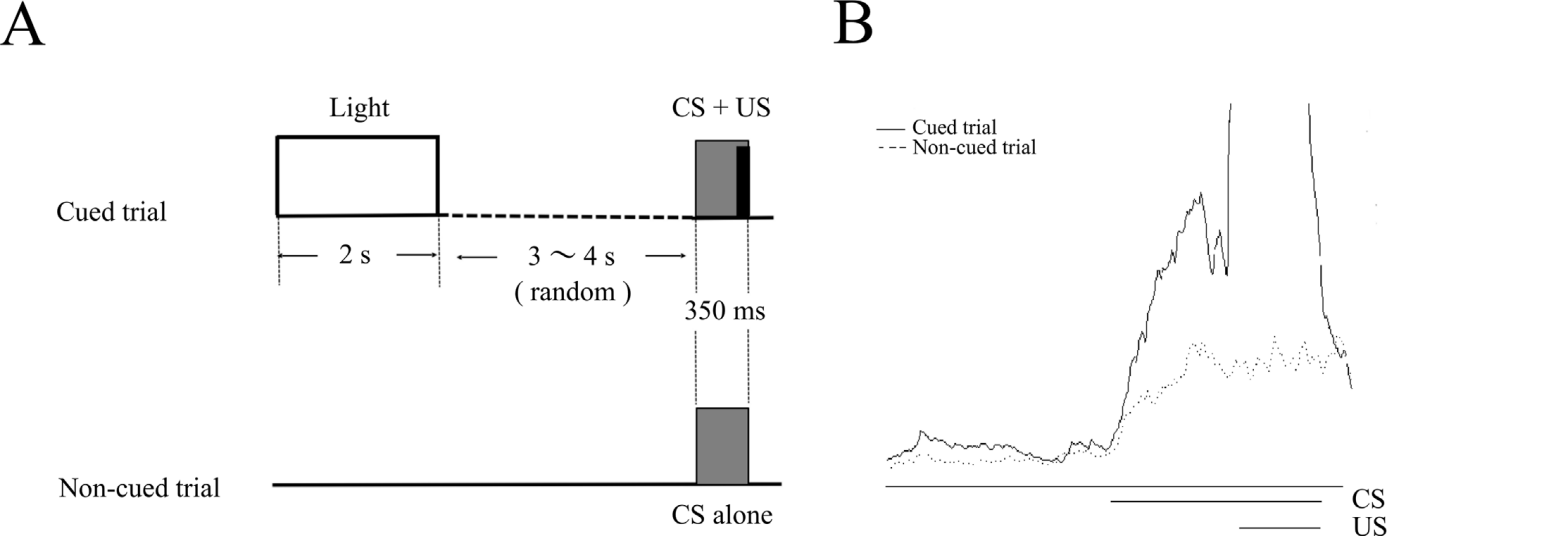
The stimulus sequence in the eyeblink serial feature-positive discrimination task. (A) Two kinds of trials were used in the task: cued trials and non-cued trials. In a cued trial, a light cue (2 s) was delivered 5 or 6s randomly before a tone conditional stimulus (CS), which co-terminated with a periorbital electrical shock unconditional stimulus (US). In a non-cued trial, the CS was presented alone, without the preceding light or US. Inter-trial intervals were randomized between 60 and 70 s. (B) The right panel shows the average of integrated EMGs in cued (black line) and non-cued trials (dotted line) of a mouse.

After at least 3 days of recovery from the surgery, each mouse was placed into an acrylic cylinder (10 cm in diameter and 23 cm in height) set in a sound- and light-attenuated chamber (53×26×31 cm). To deliver the light stimulus, 5 green wide-angle light-emitting diode chips (3 cd each) were aligned horizontally, 10 cm in width, on a side wall of the chamber at a height of 4 cm from the floor. A speaker (15 cm in diameter) was placed above the cylinder at a 40 cm height to deliver the tone CS. Mouse head was connected to a lightweight cable to deliver the US and record the EMG. During the first two days, EMG was recorded without delivering CSs and USs, so that mice could get used to the experimental apparatus (spontaneous sessions). On the second day, the mice received injections of saline or scopolamine 15 min before the start of the spontaneous session. Then, mice were conditioned for the next 7 consecutive days in the conditional discrimination task (described below) concomitant with the daily administration of saline or scopolamine, 15 min before each session (acquisition sessions). Each daily conditioning sequence consisted of 30 cued and 30 non-cued trials (Fig 1A). In a cued trial, a light cue (2 s) was delivered 5 or 6 s randomly before a tone CS (350 ms, 1 kHz, 85–87 dB, rise and fall times of 5 ms), which co-terminated with a periorbital electrical shock US (100 ms, 100 Hz). Mouse response to the US was monitored with an infrared camera and the intensity of the US (0.5–0.8 mA) was carefully adjusted to elicit an eyeblink/head-turn response. In a non-cued trial, the CS was presented alone, without the US or preceding light cue. The sequence of trials was randomized in such a way that the same type of trial was never repeated more than two consecutive times. The inter-trial intervals were randomized between 60 and 70 s. After 7 days of acquisition sessions, conditioning was continued for additional three consecutive days by switching the injected solutions, from saline to scopolamine in the control group and from scopolamine to saline in the scopolamine group, in order to examine the expression of the pre-acquired CR (expression sessions). After completing the experiment, an overdose of sodium pentobarbital (more than 100mg/kg) was administered intraperitoneally to sacrifice the mice.

### EMG analysis

The EMG activity was band-pass filtered between 0.15 and 1.0 kHz, sampled at 10 kHz, and stored on a computer hard disk. Stored EMG data were analyzed off-line using a custom-made program written in MATLAB (Mathworks, Natick, MA, USA). First, the amplitude of EMG at a given time t was calculated as the maximum difference in the raw EMG data in a time window of t ± 1 ms. Then, after eliminating the noise by setting a cutoff threshold at the level of mean ± SD over the data set of 300 ms before the CS × 60 trials, the EMG amplitude data were integrated with a 20-ms decay time constant and regarded as significant if the integrated EMG signal exceeded 30% of the threshold. Each trial was considered as valid if the frequency of occurrence of significant EMG response was less than 30% during a 200-ms pre-CS period. Among valid trials, a trial was regarded as CR containing if the frequency of occurrence of significant EMG signals was more than 30% during a 200-ms pre-US period. To evaluate the learning performance in each session, the CR percentage (CR%) was calculated by dividing the number of CR-containing trials by the number of valid trials in a session. The CR peak amplitude was calculated as the peak of the integrated EMG signal during the 200-ms interval before the US in CR-containing trials and expressed as a percentage of the threshold in the session. As an another learning index devoid of the CR judgment, an averaged value of the integrated EMG signals over the period between the CS onset and US onset was calculated and expressed as a percentage of the averaged value during 200 ms before the CS onset in each daily session.

In addition, the latency from the CS onset to the peak of the integrated EMG signal (peak latency) and the latency to the time point when the integrated EMG signal exceeded 30% of the threshold for the first time (onset latency) were calculated in CR-containing trials. In cases when there were only a few CR-containing trials in a session (less than one on average), the latency data were excluded from the analysis. Fig 1B shows an example of the response topography of a mouse.

### Statistical analyses

Statistical analyses were performed using SPSS statistical software (SPSS, Chicago, IL, USA). Data are expressed as the mean ± standard error of the mean (SEM). Differences in measured values among the groups were analyzed by the two-way analysis of variance (ANOVA) or the paired t-test. Differences were considered to be statistically significant when the P-value was less than 0.05.

## Results

### Effects of scopolamine on the acquisition of the serial feature-positive discrimination in mice

**Fig 2:**
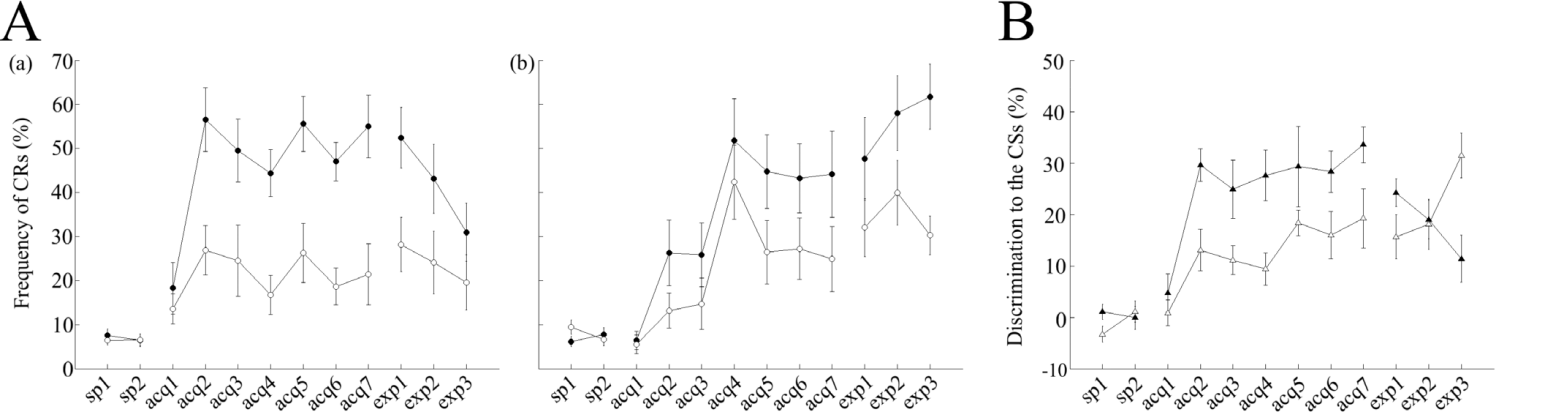
Effects of scopolamine on the acquisition and expression of the conditioned response (CR). After 2 days of adaptation sessions (sp1–2), both saline–and scopolamine–treated mice underwent acquisition sessions for 7 consecutive days (acq1–7) followed by 3 days of expression sessions (exp1–3). (A) The left and middle panels represent the averaged CR frequency in both cued and non-cued trials. (a) Averaged data from cued trials (filled circles; n = 8) and non-cued trials (empty circles; n = 8) are illustrated for saline-injected control mice. (b) Averaged data from cued trials (filled circles; n = 8) and non-cued trials (empty circles; n = 8) are shown for scopolamine-injected mice. (B) The right panel represents the discrimination (%) to the CSs between the groups. Differences in discrimination percentage between cued and non-cued trials are plotted for the saline-treated (filled triangles) and scopolamine–treated mice (empty triangles). In panel A, the vertical axis represents the CR(%) frequency while the horizontal axis illustrates corresponding sessions and the vertical bars indicate the standard error of the mean. In panel B, the vertical axis represents discrimination to the CSs(%), while the horizontal axis illustrates corresponding sessions and the vertical bars indicate the standard error of the mean. sp, spontaneous session; acq, acquisition session; exp, expression session.

During the 7 days of acquisition sessions, the saline-injected control mice learned to show a much higher number of CRs in cued trials than in non-cued trials (Fig 2Aa), despite the CS was identical in both types of trials. The CR% comprised around 50–60% (54.9 ± 8.6% on the last day of acquisition) in cued trials, whereas it remained around 20% (21.4 ± 8.4% on the last day of acquisition) in non-cued trials. A statistical comparison across sessions using a two-way ANOVA with repeated measures revealed effects of session (F(8,56) = 7.26, P<0.001) and trial type (F(1,7) = 46.36, P<0.001), as well as a significant interaction between session and trial type (F(8,56) = 8.03, P<0.001), suggesting that saline-injected control mice responded more frequently to the tone in cued trials than in non-cued trials. In addition, we have investigated differences in discrimination (difference in CR% between cued and non-cued trials) to the CSs between saline-injected control mice and scopolamine-injected mice (Fig 2B). A two-way ANOVA with repeated measures demonstrated statistically significant effects of session (F(8,56) = 12.20, P<0.0001) and groups (F(1,7) = 7.9, P = 0.026), but no significant session-group interaction (F(8,56) = 1.11, P = 0.36), suggesting that scopolamine impaired acquisition of CS discrimination between cued and non-cued trials irrespective of the cue presence.

**Fig 3:**
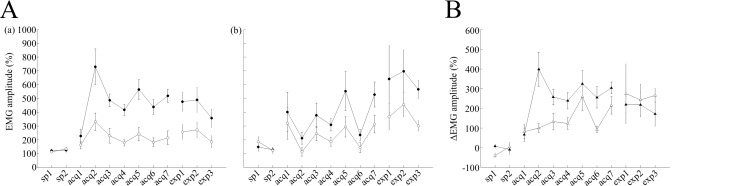
Effects of scopolamine on EMG amplitudes. (A) The left and middle panels represent the averaged EMG amplitude overall valid trials. (a) Averaged data from cued trials (filled circles; n = 8) and non-cued trials (empty circles; n = 8) are illustrated for saline-injected control mice. (b) Averaged data from cued trials (filled circles; n = 8) and non-cued trials (empty circles; n = 8) are shown for scopolamine-injected mice. (B) The right panel represents ΔEMG amplitude values between cued and non-cued trials in both groups. Filled triangles represent ΔEMG amplitude values for the saline-treated group and empty triangles represent ΔEMG amplitude values for scopolamine–treated mice. In panel A, the vertical axis represents averaged EMG amplitudes, while the horizontal axis illustrates corresponding sessions, and the vertical bars indicate the standard error of the mean. In panel B, the vertical axis represents the ΔEMG amplitude values, while the horizontal axis illustrates corresponding sessions and the vertical bars indicate the standard error of the mean.

Furthermore, we have analyzed average EMG amplitudes between the CS onset and US onset over all valid trials, which do not depend on the criterion for CR detection, and confirmed successful discrimination learning during acquisition sessions (Fig 3Aa). As in case with the CR% parameter above, two-way repeated measures ANOVA of EMG data uncovered significant effects of session (F(8,56) = 10.27, P = 0.00001) and trial type, (F(1,7) = 30.92, P = 0.001), as well as a significant interaction between session and trial type (F(8,56) = 8.76, P<0.001).

In contrast to observations in control animals, in scopolamine-injected mice, the CR% reached around 45% (44.1 ± 12% on the last day of acquisition) in cued trials, whereas it remained around 25% (24.8 ± 9% on the last day of acquisition) in non-cued trials (Fig 2Ab). A statistical comparison across the sessions using a two-way ANOVA with repeated measures revealed significant effects of session (F(8,56) = 5.62, P<0.001) and trial type (F(1,7) = 20.15, P = 0.003), as well as a significant interaction between session and trial type (F(8,56) = 4.51, P<0.001). To further confirm these results, we analyzed average EMG amplitudes over all valid trials in scopolamine-injected mice and revealed significant effects of session (F(8,56) = 2.31, P<0.05) and trial type (F(1,7) = 26.08, P = 0.001), as well as a significant interaction between session and trial type (F(8,56) = 4.79, P<0.001, Fig 3Ab). In addition, we have investigated differences in EMG patterns between the two groups (Fig 3B). A two-way repeated measures ANOVA of delta EMG amplitude (difference between EMG amplitude value in cued and non-cued trials) revealed a significant effect of session (F(8,56) = 10.23, P<0.001) and group (F(1,7) = 10.08, P = 0.016), as well as a significant interaction between session and trial type (F(8,56) = 2.63, P<0.05) indicating that group differences clearly influence the acquisition of discrimination of EMG patterns between cued and non-cued conditions in all valid trials.

### Dynamics of the CR temporal pattern

**Fig 4:**
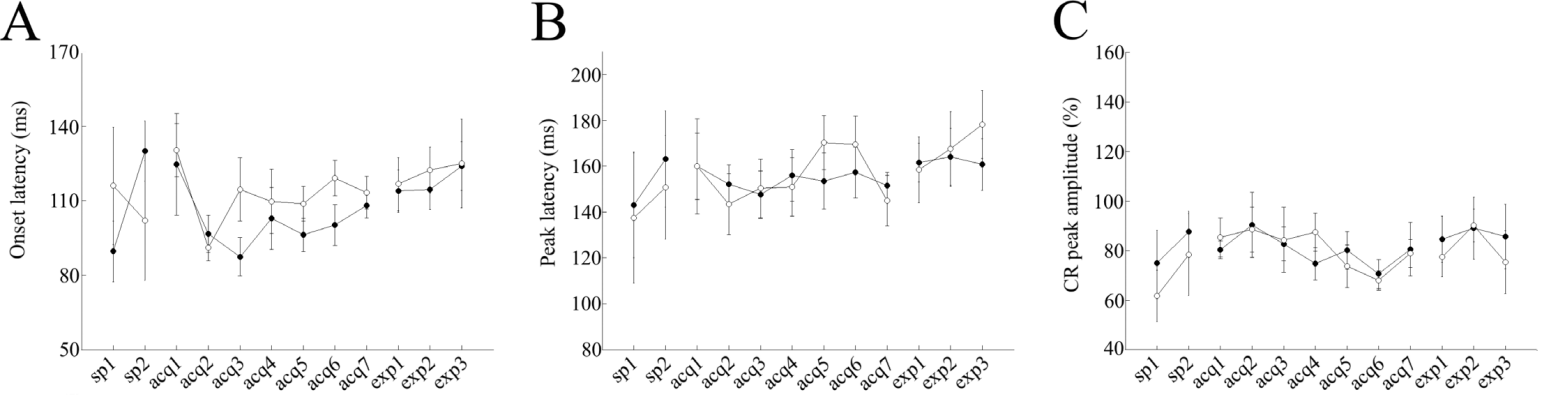
Modulation of CR dynamics in saline-treated mice. Panel (A) represents the onset latency data for cued (filled circles) and non-cued (empty circles) trials, respectively. Panel (B) represents peak latency data for cued (filled circles) and non-cued (empty circles) trials, respectively. Panel (C) represents the CR peak amplitude data for cued (filled circles) and non-cued (empty circles) trials, respectively. In panels, A and B, the vertical axis represents the average time (ms), while the horizontal axis illustrates corresponding sessions. In panel C, the vertical axis represents the CR peak amplitude (%), while horizontal axis shows corresponding sessions. Vertical bars indicate the standard error of the mean. The latencies of one control mouse in acquisition sessions 5–7 were excluded from the analysis because the average number of CR trials among non-cued trials was low (<1).

Fig 3, shows the temporal pattern of the evoked EMG activity averaged irrespectively of the CR judgment in mice of both groups during the 7 days of acquisition sessions. In agreement with observed a shorter latency of the CR onset in cued trials, the eyelid EMG pattern in saline-injected control mice showed a steeper rising phase that was clearly detected as a peak around 154 ms after the CS onset (Fig 4B). On the other hand, scopolamine-injected mice displayed a longer latency of the CR onset and CR peak in cued trials suggesting that scopolamine may change the CR temporal pattern by altering its precise CR timing via the inhibition of mAChRs.

**Fig 5:**
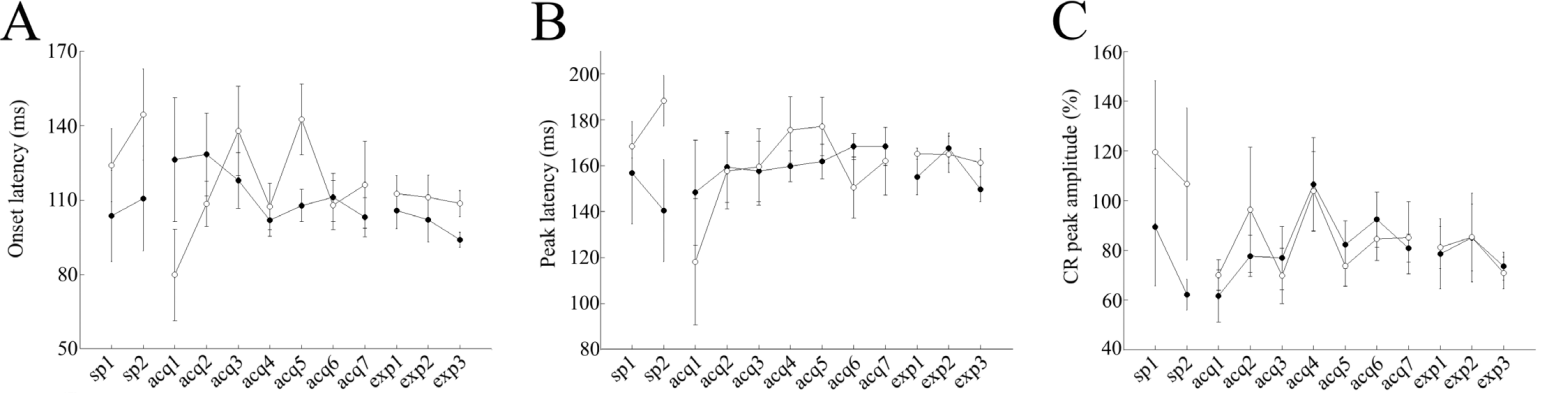
Modulation of CR dynamics in scopolamine–treated mice. Panel (A) represents the onset latency data for cued (filled circles) and non-cued (empty circles) trials, respectively. Panel (B) represents peak latency data for cued (filled circles) and non-cued (empty circles) trials, respectively. Panel (C) represents the CR peak amplitude data for cued (filled circles) and non-cued (empty circles) trials, respectively. In panels A and B, the vertical axis represents the average time (ms), while the horizontal axis illustrates corresponding sessions. In panel C, the vertical axis represents the CR peak amplitude (%), while the horizontal axis shows corresponding sessions. Vertical bars indicate the standard error of the mean.

The average CR onset latency remained around 100 and 112 ms in cued and non-cued trials, respectively, during the 7 days of conditioning in saline-injected control mice (P<0.05, paired t-test, Fig 4A). There were significant differences in the mean onset latency between cued and non-cued trials over the last 3 days of acquisition sessions (acquisitions 5–7): 101.4 ± 4.7 ms and 113.5 ± 5.1 ms in cued and non-cued trials, respectively (P = 0.007, paired t-test, Fig 4A). In contrast, the CR onset latency remained around 114 in both cued and non-cued trials, during the 7 days of conditioning in scopolamine-injected mice (P>0.05, paired t-test, Fig 5A). Additionally, there were no significant differences in the average onset latency between cued and non-cued trials over the last 3 days of acquisition sessions (107.9 ± 6 ms and 124.06 ± 7.4 ms in cued and non-cued trials, respectively; P>0.1, paired t-test, Fig 5A).

The average CR peak latency remained around 154 and 155 ms in cued and non-cued trials, respectively, during the 7 days of conditioning in saline-injected control mice (P<0.01, paired t-test, Fig 4A). There were no significant differences in the averaged peak latency between the cued and non-cued trials over the last 3 days of acquisition sessions (acquisitions 5–7): 154.1 ± 8.4 ms and 160.9 ± 10.3 ms in cued and non-cued trials, respectively (P>0.05, paired t-test). The CR peak latency remained around 163 ms and 155 ms in cued and non-cued trials, respectively, during the 7 days of conditioning in scopolamine-injected mice. Additionally, there were no significant differences in the average onset latency between cued and the non-cued trials over the last 3 days of acquisition sessions (acquisitions 5–7): 167.1 ± 5.6 ms and 155.3 ± 4.8 ms in cued and non-cued trials, respectively (P>0.1, paired t-test, Fig 5B). Also, there were no significant differences in the averaged CR peak amplitudes between the cued and non-cued trials, over the last 3 days of acquisition sessions in saline-injected control mice (acquisitions 5–7): 77.1 ± 6.6% and 75.7 ± 4.7% in cued and non-cued trials, respectively (P>0.05, paired t-test, Fig 4C). A lack of differences in averaged CR peak amplitudes was also noted in scopolamine-injected mice (85.4 ± 6.5% and 81.4 ± 5.6% in cued and non-cued trials, respectively; P>0.05, paired t-test, Fig 5C).

### Dynamics of the pre-acquired CR

Post-learning administration of scopolamine did not impair the pre-acquired CR in cued trials but led to nominally higher average CR% in non-cued trials (in cued trials: 54.9 ± 8.6% and 52.4 ± 8.4% for acquisition-7 and expression-1, respectively, and in non-cued trials: 21.4 ± 8.4% and 28.1 ± 7.5% for acquisition-7 and expression-1, respectively; P>0.05, paired t-test, Fig 2Aa). In contrast, a gradual impairment of discrimination learning was observed in expression sessions after sequential administrations of scopolamine on 3 consecutive days (in expression-1: 52.4 ± 8.4% and 28.1 ± 7.5% for cued and non-cued trials, respectively; P<0.005, paired t-test; in expression-2: 43.1 ± 9.5% and 24.1 ± 8.7% for cued and non-cued trials, respectively; P<0.01, paired t-test; in expression-3: 30.9 ± 8% and 19.6 ± 7.6% for the cued and non-cued trials, respectively; P>0.1, paired t-test, Fig 2Aa). A two-way repeated measures ANOVA across the expression sessions revealed a significant effect of trial type (F(1,7) = 26.88, P<0.05), but no effect of sessions (F(2,14) = 1.70, P>0.05). Furthermore, no significant interaction between session and trial type was detected (F(2,14) = 3.2, P>0.05) indicating that scopolamine did not impair the pre-acquired discrimination between cued and non-cued trials. On the other hand, a progressive development of discrimination learning between cued and non-cued trials was observed in scopolamine-injected mice after switching to saline injections during expression sessions (average CR% in expression-1: 47.7 ± 11.5% and 32 ± 8% for cued and non-cued trials, respectively; P>0.1, paired t-test; in expression-2: 57.9 ± 10.4% and 39.9 ± 8.9% for the cued and non-cued trials, respectively; P>0.1, paired t-test; in expression-3: 61.7 ± 9% and 30.2 ± 5.4% for cued and non-cued trials, respectively; P<0.05, paired t-test, Fig 2Ab). A two-way repeated measures ANOVA across the expression sessions detected a significant effect of trial type (F(1,7) = 18.81, P = 0.003) and a statistically significant interaction between sessions and trial type (F(2,14) = 9.29, P<0.05), while the effect of session was not statistically significant (F(2,14) = 1.02, P>0.05). These results indicate that after a switch from scopolamine to saline, mice developed discrimination learning between cued and non-cued trials. These results were further confirmed by the analysis of differences in discrimination to CSs between the groups during the expression sessions (Fig 2B). A two-way repeated measures ANOVA across the expression sessions revealed no effect of session, (F(2,14) = 33.47, P>0.05) or group, (F(1,7) = 0.05, P>0.05), but detected a statistically significant session-group interaction (F(2,14) = 11.30, P<0.05). These results suggest that there was no group effect in pre-acquired discrimination during the expression sessions. In contrast to that, factor of the group had a significant effect on the acquisition of the discrimination between cued and non-cued trials.

To further substantiate these findings, we analyzed the effects of scopolamine on the pre-acquired EMG. Post-learning administration of scopolamine did not impair the pre-acquired EMG pattern in cued trials, but led to nominal increase in average EMG amplitude values in non-cued trials, which did not reach statistical significance (cued trials: 518.6 ± 57.54% and 476.8 ± 83.2% for acquisition-7 and expression-1, respectively, non-cued trials: 215 ± 60.08% and 257.48 ± 64.55% for acquisition-7 and expression-1, respectively; P>0.05, paired t-test, Fig 3Aa). A two-way repeated measures ANOVA across the expression sessions, revealed a significant effect of trial type (F(1,7) = 11.43, P = 0.012) but no effect of session (F(2,14) = 1.08, P>0.05). The interaction between session and trial type factor was not statistically significant (F(2,14) = 0.39, P>0.05), which implied that scopolamine did not impair pre-acquired EMG pattern between cued and non-cued trials. In cued trials, EMG amplitudes in saline-injected control mice were became gradually smaller throughout the expression sessions (average EMG amplitude values in acquisition-7, expression-1, expression-1 and expression-3 were 518.6 ± 57.5%, 476.8 ± 83.2%, 490.42 ± 110.94% and 357.17 ± 79.09%, respectively, Fig 3Aa). These parameters were larger in scopolamine-injected mice (average EMG amplitude in acquisition-7, expression-1, expression-1 and expression-3 were 528.5 ± 110.8%, 641.7 ± 297.6%, 697.6 ± 190.86% and 566.77 ± 81.42%, respectively, Fig 3Ab). A two-way repeated measures ANOVA across the expression sessions in scopolamine-injected mice revealed a significant effect of trial type (F(1,7) = 7.56, P = 0.028), but no effect of session (F(2,14) = 0.36, P>0.05). The interaction between session and trial type was not significant either (F(2,14) = 0.02, P>0.05). These findings indicated that after switching from scopolamine to saline, mice exhibited different EMG amplitudes between cued and non-cued trials. In accordance with these results, delta EMG amplitude values became gradually smaller in saline-injected control mice (303%, 219%, 218%, and 171% in acquisition-7, expression-1, expression-2 and expression-3, respectively, Fig 3B). Furthermore, was also consistent with the trend of gradually decreasing differences in CR% to CSs (33%, 24%, 19%, and 11% in acquisition-7, expression-1, expression-2, and expression-3, respectively, Fig 2B), throughout the expression sessions. On the other hand, delta EMG amplitude values became gradually larger (214%, 274%, 242%, and 265% in acquisition-7, expression-1, expression-2 and expression-3, respectively, Fig 3B) in scopolamine-injected mice, which was consistent with gradual increasing differences in CR% to the CSs throughout the expression sessions (19%, 15%, 18%, and 31% in acquisition-7, expression-1,expression-2 and expression-3, respectively, Fig 2B). These results were further confirmed by the statistical analysis of delta EMG amplitude values in the two groups during the expression sessions. A two-way repeated measures ANOVA failed to detect statistically significant effects of group (F(1,7) = 0.22, P>0.05) or session, (F(2,14) = 0.08, P>0.05), despite a significant effect of group observed during the acquisition sessions. The session-group interaction during the expression sessions also lacked statistical significance (F(2,14) = 0.09, P>0.05).

Although saline-injected control mice did not show any significant differences in the averaged onset latency between cued and non-cued trails (P>0.1, paired t- test), the latency of the CR onset in cued trials gradually became longer throughout the expression sessions (108 ± 5.1 ms, 113.8 ± 8.5 ms, 114.4 ± 8, and 123.9 ± 9.7 ms in acquisition-7, expression-1, expression-2 and expression-3, respectively, Fig 4A). In contrast, the latency of the CR onset in cued trials became gradually shorter throughout the expression sessions in scopolamine–treated mice (103.2 ± 7.8 ms, 105.8 ± 7.2 ms, 102 ± 8.9 ms and 94 ± 2.9 ms in acquisition-7, expression-1, expression-2 and expression-3, respectively, Fig 5A). Additionally, on the third day of expression sessions, the CR onset latency was significantly different between cued and non-cued trials (94 ± 2.9 ms and 108.6 ± 5.2 ms, in cued and non-cued trials, respectively, P<0.05, paired t-test, Figs 3B and 4B). In addition, in both treatment groups, there were no significant differences in the peak latency and CR peak amplitudes between cued and non-cued trials in the expression sessions (P>0.05, paired t-test, Figs 4B, 4C, 5B and 5C).

## Discussion

We investigated the role of mAChRs in serial feature-positive discrimination in eyeblink conditioning by treating mice with the mAChR antagonist scopolamine. We found that control mice successfully acquired the discrimination between cued and non-cued trials, whereas scopolamine-injected mice exhibited indistinguishable CRs in both trials. Administration of scopolamine after successful learning did not disturb pre-acquired discrimination or the CR expression itself. These results suggest that mAChRs may play an important role in the acquisition of discrimination between cued and non-cued trials but not in mediating discrimination itself after the conditional memory had formed in the serial feature-positive discrimination task.

### Serial feature-positive discrimination in mouse eyeblink conditioning

In the present study, control mice successfully acquired the discriminative response to the identical tone CS based on the presence/absence of preceding light stimuli: the frequency of the CR occurrence in cued trials was much higher than that in non-cued trials (Fig 2A). We used the serial feature-positive discrimination eyeblink conditioning task with a temporal gap of 3–4 s between the end of the conditional cue and the CS onset, which corresponded to an inter-stimulus interval of 5–6 s between their onsets. This temporal relationship was almost equal to 3–5-s gap (5–7-s interval) used in rat serial feature-positive discrimination [22] and 3.4-s gap (4.2-s interval) required for the emergence of occasion setting in the serial conditional discrimination in rabbit [18]. At the same time, a much shorter gap of 1 s (5-s interval) [15] or even an overlap of the feature and target (3.3-s interval) [16] could be sufficient to detect the impairment of amnesic patients in the serial conditional discrimination. It should be noted that in rabbit serial conditional discrimination with a temporal gap of less than 3.4 s (4.2-s feature-target interval and 4.6-s feature-US interval), the control over the response to the target CS by the feature was weak and instead, a direct response to the feature was noted [18]. Consistent with the importance of the temporal gap for the top-down modulation of the eyeblink CR in animals, the temporal gap between the feature and target stimuli enhanced the occasion setting ability of the feature stimulus in the feature-positive discrimination in rat appetitive conditioning [33]. Therefore, the temporal gap or long feature-target interval may be important for establishing the top-down modulation of the CR in both eyeblink and appetitive conditioning tasks.

In addition to the difference in the frequency of the CR occurrence, mice exhibited differentially timed CR depending on the presence/absence of the preceding cue: the CR onset latency was shorter in cued trials than in non-cued trials (Fig 4A), although CR peak amplitude values were not significantly different between trial types (Fig 4C). This similarity in CR peak amplitudes indicates that once the CR had been triggered, it fully developed irrespective of the trial type, suggesting that the top-down modulation mainly occurred before the execution of the CR, leading to the difference in its frequency of occurrence, but not during the CR itself. This notion is consistent with the earlier onset of CR in cued trials reflecting an increased level of attention to the coming CS by the light cue, which might serve to gate the input-output relationship in the cerebellum and related brain stem structures. Similarly, in human conditional discrimination eyeblink conditioning, there was a nominal tendency towards shorter CR onset latency values in reinforced trials compared to non-reinforced trials, although the difference did not reach statistical significance [16]. In addition, other studies, which investigated the top-down modulation of the startle reflex, reported that the attention or awareness to the coming stimulus caused shorter latency responses [34]. Therefore, the earlier CR onset in cued trials might reflect a top-down modulation process initiated by the preceding cue.

### Involvement of mAChRs in the serial feature-positive discrimination

We found that scopolamine impaired the acquisition of the conditional discrimination in the serial feature-positive discrimination task, as drug-treated mice manifested CRs to an equal degree in both the cued and non-cued trials (Fig 2Ab). Therefore, the acquisition of the CR itself was not appreciably impaired. These results suggested that scopolamine impairs the formation of the memory for the top-down modulation, but not the acquisition of the CR itself. The latter process largely depends on the activity of the cerebellum. In addition, scopolamine did not significantly impair pre-acquired conditional discrimination performance and expression of the CR itself when tested in control mice after 7 days of acquisition sessions (Figs 2A, 3A and 3B). These results paralleled the weak effect of scopolamine on the pre-acquired CR in the hippocampus-dependent trace eyeblink conditioning in rabbits [29]. Therefore, mAChRs might play an important role in the formation, but not the expression, of the memory for top-down modulation associated with the feature cue in the serial feature-positive discrimination task in mouse eyeblink conditioning.

In addition to affecting the response probability, scopolamine influenced CR temporal pattern as scopolamine-injected mice showed the equivalent CR onset latency in cued and non-cued trials (Fig 5A), while saline-injected mice displayed a shorter latency of the CR in cued trials than in non-cued trials (Fig 4A). Similarly, in human eyeblink conditional discrimination task, the patients with medial temporal lobe amnesia exhibited a similarly timed CR in reinforced and non-reinforced trials, while control subjects displayed differently timed CRs with a longer CR duration and a tendency to an earlier CR onset [16]. These findings suggest an involvement of mAChRs in refining CR timing in serial feature-positive discrimination learning.

Because the detrimental effect of scopolamine on the learning rate of delay eyeblink conditioning was abolished by hippocampal ablation in rabbits [35], the hippocampus and related brain areas are the most plausible scopolamine targets, which mediate effects of the drug on the conditional discrimination that uses delay eyeblink conditioning. This notion is supported by numerous studies that demonstrated the involvement of the hippocampus in conditional discrimination learning [15, 16, 22]. Furthermore, the effects of scopolamine in the present study are consistent with previous observations of its ability to severely impair hippocampus-dependent trace eyeblink conditioning [29] but not delay eyeblink conditioning in mice [30], or only mildly affected in rabbits [29]. Involvement of the hippocampus in the appetitive serial feature-positive discrimination has been proposed, as the ablation of the hippocampus and associated neural pathways prevented the acquisition of discrimination and eliminated the retention of the pre-acquired discrimination [36]. However, neurotoxic hippocampal lesions that specifically destroyed hippocampal neurons failed to cause any impairment. This finding indicated the involvement of other structures other than the hippocampus [37–39]. Therefore, there remains a possibility that extra-hippocampal regions, which express mAChRs, play an important role in the serial feature-positive discrimination task in mouse eyeblink conditioning.

## Conclusions

We have shown that mAChRs play a pivotal role in the acquisition of discrimination between cued and non-cued trials as pharmacological inhibition of their activity resulted in an indistinguishable expression of CRs in both trials. On the other hand, the administration of scopolamine did not disturb pre-acquired discrimination performance or expression of the CR itself. In addition, injections of scopolamine led to similar CR dynamics in both trials. Collectively, these results, suggest that mAChRs may play an important role in the formation, but not expression, of the memory for top-down modulation in the serial feature-positive discrimination task in mouse eyeblink conditioning. Since there are 5 different subtypes of mAChRs with different roles in learning, in the future, we need to investigate the role of individual mAChR sub-type in this type of conditioning.
